# Near Visual Acuity Following Hyperopic Photorefractive Keratectomy in a Presbyopic Age Group

**DOI:** 10.5402/2012/310474

**Published:** 2012-05-20

**Authors:** Michael Moore, Antonio Leccisotti, Claire Grills, Tara C. B. Moore

**Affiliations:** ^1^School of Biomedical Sciences, University of Ulster, Coleraine BT52 1SA, UK; ^2^Department of Ophthalmology, Casa di Cura Rugani, Piazza Giovanni Amendola 11, 53100 Siena, Italy

## Abstract

*Purpose*. To assess near visual acuity in a presbyopic age group following hyperopic photorefractive keratectomy (PRK). *Setting*. Private practice in Siena, Italy. *Methods*. In this retrospective single-surgeon comparative study, PRK with mitomycin C was performed to correct hyperopia using Bausch & Lomb 217z laser for 120 eyes of 60 patients in the presbyopic age group (mean spherical equivalent SE +2.38 D ± 0.71 D and mean age 52 ± 5.09). 120 eyes of 60 age-matched controls (mean age 54 ± 5.09) had their unaided near vision measured. *Results*. At 12 months mean SE was −0.10 D ± 0.27 D in the PRK group. Mean best corrected visual acuity (BSCVA) was 0.005 ± 0.022  log MAR; 2 eyes lost ≥0.1 log MAR. Mean uncorrected visual acuity was 0.04 ± 0.077  log MAR. Mean distance corrected near visual acuity (DCNVA) in the PRK group was *J*3.73 ± 1.06. This was statistically better (*P* < 0.05) than the mean unaided near visual acuity in the control group *J*4.07 ± 1.08. *Conclusion*. PRK was found to be safe, predictable, and an effective way of correcting hyperopia in this age group. It was also found to give better than expected near vision.

## 1. Introduction

Photorefractive keratectomy (PRK) has been a common method for the correction of refractive error for some time [1–3]. Although there is some debate as to the level of hyperopia correctable with PRK, PRK is seen as a safe and reliable method for the correction of low to moderate amounts of hyperopia [4].

Presbyopia is an on-going challenge for the refractive surgeon, and, as of yet, no single surgical solution has been found. It has been observed in some cases that, following corneal refractive surgery, presbyopic patients appear to develop pseudoaccommodation and have better than expected near visual acuity [5–7]. It has been suggested that changes in corneal topography and ocular aberrations following both PRK and radial keratotomy (RK) are responsible for this finding [5, 7, 8]. An increase in ocular aberrations may cause an increase in the depth of field (DOF) and allow for better than expected near vision in the presbyopic age group.

Currently, there is no literature detailing the level of near visual acuity following hyperopic PRK (MEDLINE search with key words “photorefractive keratectomy,” “PRK,” “presbyopia,” “near vision,” “near visual acuity”). A previous study [7] assessing the level of near vision following myopic PRK in presbyopes has been conducted; however, this study contained a relatively small sample size of 10 eyes. Felipe et al. have described PRK treatment to treat presbyopia [9].

This study evaluated the distance and near vision (with best corrected distance vision) in a group of 120 presbyopic posthyperopic PRK eyes with age-matched controls assessing for evidence of the safety, efficacy, and predictability of performing hyperopic PRK on presbyopic eyes.

## 2. Patients and Methods

This retrospective comparative single surgeon (AL) study included presbyopic eyes undergoing hyperopic PRK to correct distance vision with a target postoperative refraction of plano. It also included a control group which received no treatment whatsoever. All treatments were performed in a private practice in Siena, Italy. All patients signed a consent form for hyperopic correction by PRK.

 Inclusion criteria were as follows.

- An age between 45 and 65.
- A cycloplegic refraction with spherical equivalent between +1.00 and +5.00.
- Refractive stability for the last 2 years.
- Absence of any previous corneal or lens surgery.
- Absence of any corneal, macular, inflammatory, or lens abnormality.
- Absence of collagen disease or diabetes.
- Ultrasound central corneal pachymetry greater than 500 microns.
- Regular corneal topography with no signs of contact lens induced warpage and a central curvature less than 45 dioptres.
- Keratoconus was ruled out by a combination of topography indexes and pattern, and pachymetry as detailed in angle-supported phakic intraocular lenses in eyes with keratoconus and myopia [10].
- Best spectacle corrected visual acuity (BSCVA) of 0.2 log MAR units was required for an eye to be included.
- Absence of pseudophakia.

Followup for assessment of near and distance visual acuity was 12 months following the treatment.

### 2.1. Preoperative Examination

 Preoperative assessment consisted of

- uncorrected visual acuity (UCVA),
- BSCVA,
- autorefractometry assessed with Retinomax 2 (Nidek),
- manifest and cycloplegic refraction (by cyclopentolate),
- undilated and dilated slit lamp evaluation,
- axial and tangential corneal topography assessed with Oculus Keratograph (Iculus Wetzlar, Germany),
- tonometry assessed with Goldmann tonometer,
- dilated funduscopy.

Soft contact lens use was interrupted 1 month before examination and surgery, while rigid contact lens use was interrupted 3 months before examination and surgery.

### 2.2. Surgical Technique

A Bausch & Lomb Technolas 217z excimer laser was used in Planoscan mode. A 6 mm optical zone was chosen in all eyes. The cycloplegic spherical error was fully treated. The left eye was treated immediately after the right. Laser fluence was calibrated obtaining a fully red area (with tiny aluminum remnants) on the calibration plate with 65 spots. No nomogram adjustments were used.

Before PRK, topical anesthesia comprising 3 to 5 drops of oxybuprocaine 0.4% was administered. After an eyelid speculum was inserted, manual deep ithelialization was performed in a 10.0 mm circular area with a blunt golf-club spatula and the epithelium was discarded. Laser ablation was centred on the visual axis, which was identified by the superimposed Purkinje images.

Ten mL of BSS at 10°C was dripped on the cornea, and the cornea dried by a Merocel sponge. Another Merocel sponge soaked with mitomycin C 0.2 mg/mL (corresponding to 0.02%) (Kyowa Hakko Kogyo Co. Ltd., Tokyo, Japan) was applied on the stromal bed for 45 seconds. Finally, the stromal bed was irrigated with 30 mL of BSS at 10°C. Topical levofloxacin 0.3% and diclofenac were instilled, and a balafilcon a bandage contact lens (Pure Vision, Bausch & Lomb) was applied.

### 2.3. Postoperative Examinations

All patients had postoperative examinations at 2, 3, 4, 6, and 30 days as well as 2, 4, 6, and 12 months. A slit lamp examination was performed at all examinations. Manifest BSCVA, UCVA, and tonometry were measured at all visits beginning at 30 days. At the 12 month visit, unaided near visual acuity was assessed. It was not assessed at any other stage in order to remove any possible learning effects. Near visual acuity was also assessed with any distance correction required to negate the effects of under or over correction.

### 2.4. Near Vision

Following the surgery, an assessment of near vision was carried out for all eyes. This assessment was carried out by the same person (AL), with the same reading chart and in the same lighting conditions to ensure no extraneous factors influenced the results. Any eyes where the spherical equivalent was not 0.00 following the PRK had their near vision assessed with their required distance refraction in place, so as to negate any effects of over or under correction. All eyes with a spherical equivalent of 0.00 had their near vision assessed unaided. Near vision was measured using Jaeger notation.

At this stage, the control group had their unaided near visual acuity measured as a comparison.

### 2.5. Statistical Analysis

Statistical analysis was performed by PASW Statistics v.18 (SPSS: An IBM Company). The unit of data analysis used was per eye. An independent *t*-test was performed for the PRK and control groups.

## 3. Results

### 3.1. Preoperative Data

A total of 120 eyes of 60 patients passed the inclusion criteria and gave consent to be included in this study. All eyes had hyperopic PRK to correct distance refractive error and were in an age group affected by presbyopia. A group of age-matched healthy presbyopic emmetropes (cycloplegic refraction in each eye comprised between +0.25 and −0.25 D) was used as a control group. All patients attended for a 12-month followup.

All PRK group patients were in the presbyopic age group with a mean age of 52 and range of 45 to 65 (SD 5.09). The mean age for the control group was 54 with a range of 45 to 65 (SD 5.09). The difference in age was not statistically significant (*P* = 0.900).

Preoperatively, the mean spherical equivalent (SE) of the PRK group was +2.38 with a range of +1.00 to +4.75 (SD 0.71). Preoperatively, the mean BSCVA of the PRK group was 0.003 Log MAR with a range of 0.00 to 0.10 log MAR (SD 0.015).

### 3.2. Predictability

Predictability was measured by mean SE at 12 months. Postoperatively, the mean SE was −0.10 with a range of +0.25 to −1.00 (SD 0.27). At 12 months, 109 eyes (91%) were within ±0.50 D of the intended correction and 120 eyes (100%) were within ±1.00 D of the intended correction.

### 3.3. Safety

**Figure 1:**
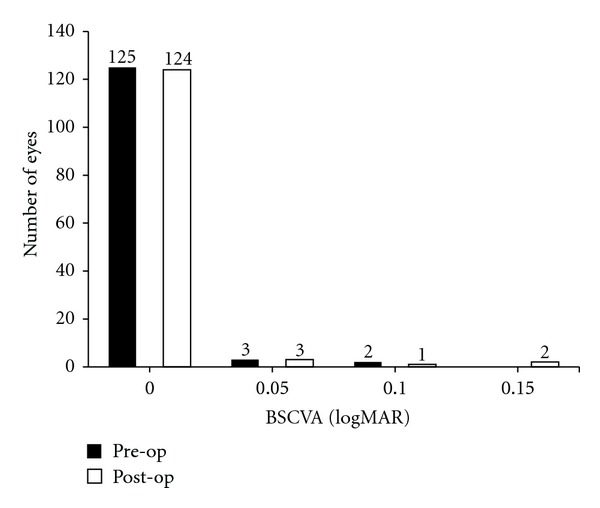
This figure shows pre- and postoperative best spectacle corrected visual acuity (BSCVA) for 120 presbyopic eyes undergoing hyperopic PRK. The operation had a safety index of 0.995 with 2 eyes losing ≥0.1 log MAR and all other eyes maintaining or having an increase in BSCVA.

Safety was evaluated by changes in BSCVA, observed at 12 months. In the PRK group, mean BSCVA at 12 months was 0.005 log MAR with a range of 0.00 to 0.15 (SD 0.022) log MAR. 2 eyes lost ≥0.1 log MAR acuity with all other eyes maintaining or seeing an increase in BSCVA as seen in Figure 1. The safety index (postoperative BSCVA/preoperative BSCVA) was 0.995.

### 3.4. Efficacy

Efficacy was evaluated by UCVA at 12 months. The mean UCVA that was at 12 months was 0.04 (SD 0.077) log MAR with a range of 0.00 to 0.30 log MAR. Efficacy index (postoperative UCVA/preoperative BSCVA) was 0.92.

### 3.5. Near Vision

**Figure 2:**
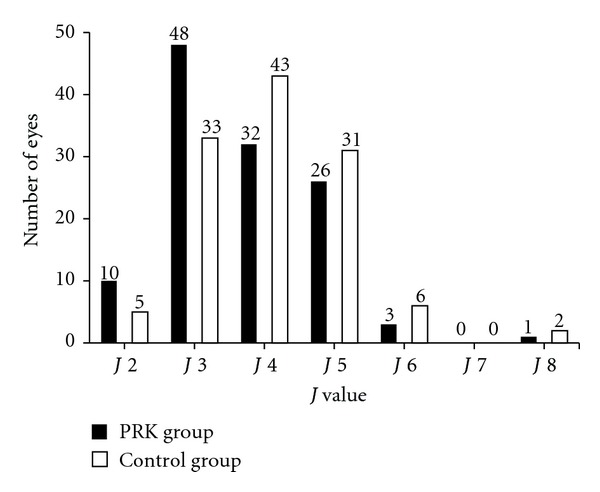
This figure compares the distance corrected near visual acuity (DCNVA) of 120 posthyperopic PRK eyes, which are also presbyopic (PRK group), and the uncorrected near visual acuity (UNVA) of 120 emmetropic presbyopic eyes (control group). The near visual acuity was found to be better to a statistically significant extent (*P* < 0.05) in the PRK group. The PRK group was shown to have a higher percentage of eyes with better NVA. Almost 50% of the PRK eyes had NVA of *J* 3 or better and 75% had NVA of *J* 4 or better.

Figure 2 summarizes the *J* values found for the two groups. The mean *J* value for the PRK group was *J* 3.73 with a range of *J* 2 to *J* 8 (SD 1.06). The mean *J* value for the control group was *J* 4.07 with a range of *J* 2 to *J* 8 (SD 1.08). An independent *t*-test was performed which showed the near visual acuity was better in the PRK group to a statistically significant extent (*P* < 0.05). The *t*-test was repeated to compare right and left eyes separately and again showed a statically significant (*P* < 0.05) difference between the two groups with the right and left eyes of the PRK group having lower *J* values than the control group. The right and left eyes were also compared for each group with the mean for the right PRK eyes *J* 3.68 and the left PRK eyes *J* 3.78. The control group also showed slightly better near visual acuity in the right eyes, with right eyes having a *J* value of 4.02 and left eyes a value of *J* 4.12. In neither group was the difference between right and left eyes statistically significant.

Almost 50% of the PRK group had a *J* value of 3 or better and 75% had a *J* value of 4 or better. There are also twice as many eyes with a *J* value of 2 in the PRK group then are present in the control group. The mode is also lower in the PRK group with a *J* value of 3, whereas the modal *J* value for the control group is 4 (Table 1).

### 3.6. Complications

All eyes selected for this study underwent uneventful PRK with no complications occurring.

## 4. Discussion

This study found PRK to be a safe, predictable, and effective way of correcting low to moderate levels of hyperopia in a presbyopic age group. Hyperopia is the most common refractive error [11] in human eyes, and its incidence increases with age [1]. PRK has previously been shown to be an effective way of correcting low to moderate levels of hyperopia [2–4, 12]. There is however some debate of the level of hyperopia which can be corrected [1]. It has been asserted that higher age groups may make PRK corrections less predictable [13]. Data from our study, presented here, would seem to agree with previously published data by O'Brart et al. [4], who also had an older age group for their study and found no evidence of hyperopic shift or late regression 7.5 years after hyperopic PRK. However, our study only includes data at up to a 12-month followup. In a similar study, Ghanem et al. [14] found that LASIK posed no greater risk of visual loss in older age groups.

 In this study, the post-PRK eyes were observed to have significantly better near acuity than would be expected for presbyopic eyes, when compared to a control group which has had no surgery.

It may be that this better than expected near vision is due to an increase in depth of focus (DOF). Depth of focus (DOF) can be defined as “the distance in front and behind the focal point over which the image may be focused without causing a sharpness reduction” [15] for a given optical system. The depth of field is the projection of the DOF into object space.

DOF is affected by several factors which can be separated into two categories; external or internal. External factors include luminance, contrast, wavelength of light, spatial frequency, and target detail [16]. Internal factors include visual acuity, pupil size, accommodation, retinal eccentricity, ocular aberrations, and age [16]. By altering any of these conditions, the DOF will either increase or decrease. If DOF was sufficiently increased, it should reduce the effects of presbyopia. This means that if ocular aberrations were to increase, the effects of presbyopia could be reduced.

A well-observed complication following corneal refractive surgery is an increase in higher-order aberrations [17]. Tanabe et al. [18] showed a link between increased higher order aberrations (HOAs) and reduced low contrast visual acuity following PRK. They found the most prevalent HOAs to be spherical aberration (SA) and coma. Their results were echoed by Oshika et al. [19] who also found large increases in SA and coma following myopic PRK and LASIK.

Bakaraju et al. [20] conducted an experiment using model eyes to assess the effect of positive and negative spherical aberration (SA) on DOF. The authors found that for higher levels of negative SA, a larger DOF was observed. They also found that, for lower levels, both negative and positive SA increased the DOF. This suggests that following PRK in our group of eyes, there would be increased negative SA which, according to Bakaraju et al. [20], should lead to an increase in DOF. Rocha et al. [21] conducted a similar study which also showed that inducing spherical aberration can expand the depth of focus of a human eye. This increase in the DOF is a possible explanation for the reduced effect of presbyopia in our post-PRK eyes. A comparison of pre- and postoperative SA of these eyes could have confirmed a relationship between improved near vision and SA, but due to the retrospective nature of this study, this information could not be obtained.

This association between SA and DOF in presbyopes was also investigated by Rocha et al. [22], who compared the level of distance corrected near and intermediate vision in patients who had either aspheric (AcrySof IQ, Sensar AR40) or spherical (AcrySof SN60AT) intraocular lenses implanted during cataract surgery. Patients implanted with a spherical intra-ocular lens were found to have higher levels of SA but also had significantly better levels of distance corrected near visual acuity. The apparent pseudoaccommodation of the spherical intraocular lens group was also found to be 0.4 D higher than the aspherical intraocular lens group. They also found that the only HOA which was statistically different between the three types of lenses implanted was SA with the spherical intraocular lens inducing higher levels of positive SA. The aspherical lenses did however have better in-focus visual performance.

The only other study which assessed the level of near vision in presbyopes following a laser corneal refractive procedure was conducted by Artola et al. [7]. They assessed 10 myopic eyes following PRK and found they had improved near visual acuity which they attributed to an increase in corneal spherical aberration. They found that 6 of the 10 eyes which had PRK were found to have near visual acuity of *J* 1+. According to Bakaraju et al. [20], it would be expected that the level of near acuity would be better in our study as hyperopic PRK was performed which would induce negative SA which should give better near vision. This difference may be explained by the small sample size in the study by Artola et al. [7].

Although multifocal corneal laser surgery has not become widely excepted [23], one of the most commonly performed variations is with the ablation profile global optimum [24]. This creates a hyperprolate cornea to aid near vision while trying to have a minimal impact on the distance vision. This is likely quite similar to what is occurring in the PRK eyes in this series.

A lot of current research in corneal laser refractive surgery relates to minimising any surgically induced higher order aberrations following surgery such as SA [25–27]. Our study shows that unaided near visual acuity is better than expected following hyperopic PRK. It is believed this is due to surgically induced higher-order aberrations. Future corneal refractive surgery to correct presbyopia may be able to make use of these findings to improve multifocal corneal ablation profiles.

## Figures and Tables

**Table 1:** Summary of near visual acuity (NVA) for PRK and control group.

	PRK group	Control group
Number of eyes	120	120
Mean NVA	*J* 3.733	*J* 4.067
Median NVA	*J* 4	*J* 4
Mode NVA	*J* 3	*J* 4

NVA = DCNVA for PRK group and UCVA for control group.

This table shows the main NVA findings. The mean NVA following PRK was found to be better to a statistically significant extent (*P *<0.05). The most common level of NVA found in each group was also better in the PRK group.
